# Methamphetamine Enantiomers: From Chemical Synthesis to Biological Fate

**DOI:** 10.1002/chir.70136

**Published:** 2026-08-13

**Authors:** Larissa Pires do Espírito Santo, Renato Lúcio de Carvalho, Juliana Almeida Sad, Pedro Henrique Costa dos Santos, Rhaiza Silva Domingues, Gabriele de Azevedo Cardoso, Karine Braga Enes, Gisele Assis Castro Goulart, Maila de Castro Lourenço das Neves, Ângelo de Fátima

**Affiliations:** ^1^ Department of Chemistry, Institute of Exact Sciences Universidade Federal de Minas Gerais Belo Horizonte MG Brazil; ^2^ Pós‐graduação em Inovação Tecnológica, Department of Chemistry Universidade Federal de Minas Gerais Belo Horizonte MG Brazil; ^3^ Pós‐graduação em Química, Department of Chemistry Universidade Federal de Minas Gerais Belo Horizonte MG Brazil; ^4^ Department of Pharmaceutics, Faculty of Pharmacy Universidade Federal de Minas Gerais Belo Horizonte MG Brazil; ^5^ Pós‐graduação em Ciências Farmacêuticas, Faculty of Pharmacy Universidade Federal de Minas Gerais Belo Horizonte MG Brazil; ^6^ Department of Psychiatry, Faculty of Medicine Universidade Federal de Minas Gerais Belo Horizonte MG Brazil; ^7^ Department of Organic Chemistry, Faculty of Science Atatürk University Erzurum Türkiye

**Keywords:** biological behavior, enantioselectivity, methamphetamine, organic synthesis, pharmacodynamics, pharmacokinetics

## Abstract

Methamphetamine, an amphetamine‐type stimulant, is a potent and psychoactive compound distinguished by the presence of a single stereogenic center in its structure. Consequently, it exists as two enantiomers: (*S*)‐(+)‐methamphetamine [(*S*)‐(+)‐MA (**1**)] and (*R*)‐(−)‐methamphetamine [(*R*)‐(−)‐MA (**2**)]. The chirality of these molecules confers distinct biological and pharmacological properties, with notable differences between the two forms. These enantioselective variations extend beyond their mechanisms of action, encompassing differences in the central nervous system interactions, metabolism, excretion, and other physiological processes. Understanding these disparities is crucial for elucidating the pharmacological and potential therapeutic profiles of each enantiomer. Achieving such insight requires examination of the enantioselective synthetic routes through which the desired chirality can be either inherited or constructed. This review integrates chemical and biological perspectives to provide a comprehensive overview of both methamphetamine enantiomers, thereby bridging the gap between laboratory synthesis and biological behavior in these two chemically fascinating yet dangerous molecules.

## Graphical abstract

Methamphetamine is an amphetamine‐type stimulant characterized by a single stereogenic center, resulting in two enantiomers, (*R*)‐(−)‐methamphetamine and (*S*)‐(+)‐methamphetamine, with distinct biological and pharmacological profiles. These forms exhibit differences in central nervous system activity, metabolism, excretion, and overall physiological responses. This review combines chemical synthesis and biological insights to emphasize how enantioselectivity influences their pharmacological and toxicological behavior.

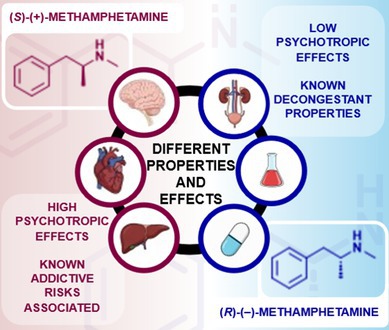

**Abbreviations**
ADHD: deficit hyperactivity disorder
AT: (*S*)‐(+)‐methamphetamine [(*S*)‐(−)‐MA (**1**)], (*R*)‐(−)‐methamphetamine [(*R*)‐(−)‐MA (**2**)], amphetamine
AT: amphetamine
ATS: amphetamine‐type substances
BBB: blood–brain barrier
CNS: central nervous system
CSP‐LC–MS/MS: chiral stationary phase coupled with tandem mass spectrometry
DA: dopamine
DAT: dopamine transporter
FDA: Food and Drug Administration
GC/MS‐NICI: gas chromatography coupled with mass spectrometry in negative ion chemical ionization mode
GC–MS: chromatography–mass spectrometry
HHS: Health and Human Services
HPLC: high‐performance liquid chromatography
HPLC‐ESI‐MS: liquid chromatography coupled with electrospray ionization mass spectrometry
IAC: immunoaffinity chromatography
LC–MS/MS: chromatography–tandem mass spectrometry
L‐TPC: *N*‐trifluoroacetyl‐*L*‐prolyl chloride
MA: methamphetamine
MAO‐B: monoamine oxidase B
MOFs: metal–organic framework
NAc: nucleus accumbens
NE: norepinephrine
NET: norepinephrine transporter
PET: positron emission tomography
SERT: serotonin
VMAT‐2: supercritical fluid extraction (SFE) and vesicular monoamine transporter‐2

## 1 Introduction

Amphetamine‐type substances (ATS) are among the most widely used recreational drugs [1], representing a clear public health concern [2]. According to the World Drug Report 2023, amphetamine (AT) and methamphetamine (MA), the two main principal members of this class, were the third most commonly used drugs worldwide in 2021, with an estimated 36 million users [1, 3].

**FIGURE 1:**
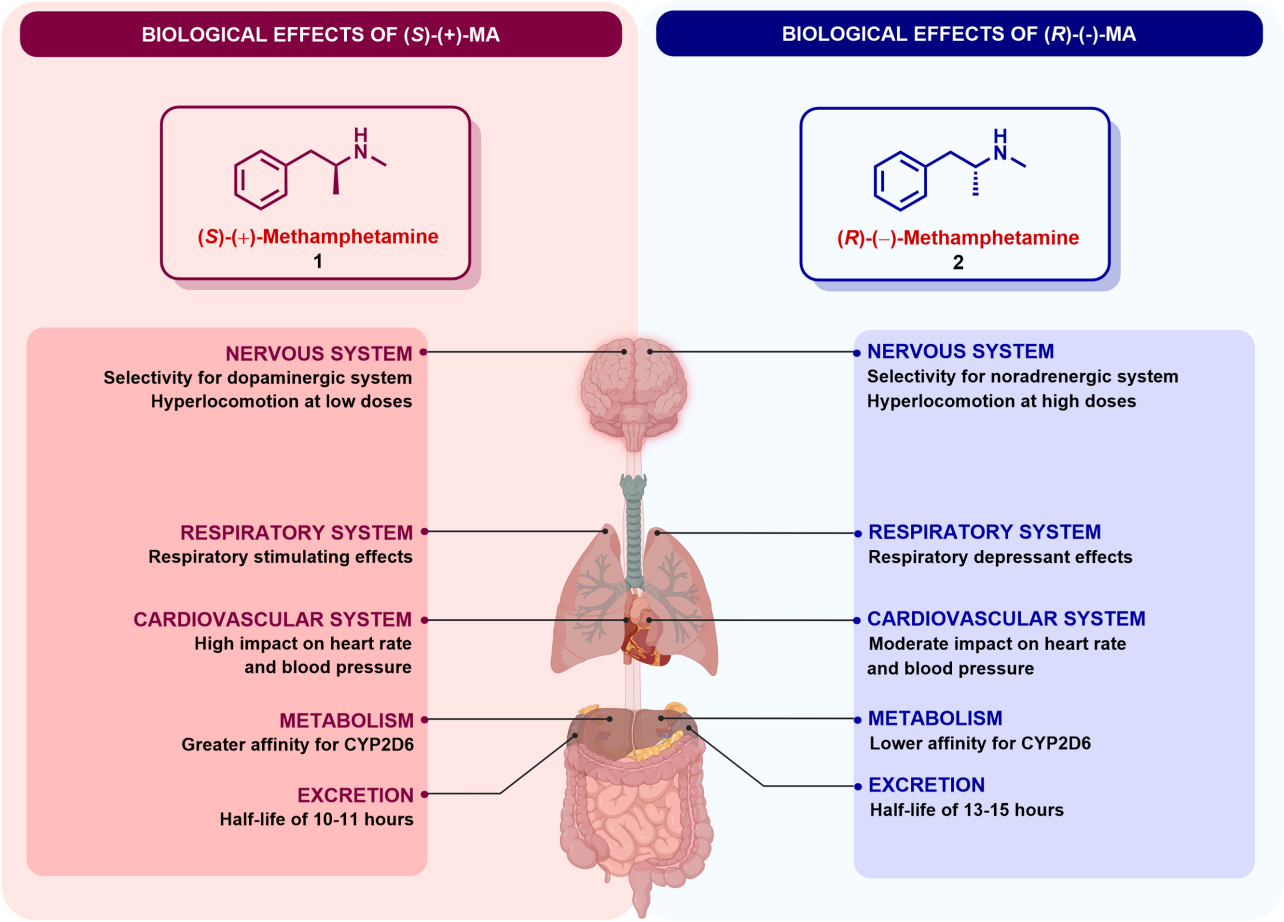
Biological effects of both (S)‐(+)‐methamphetamine **(1)** and (*R*)‐(−)‐methamphetamine **(2)** in human systems (Image created using Biorender.com).

In the context of illicit use, MA is commonly known as “meth,” “speed,” or “shabu” and represents the predominant ATS on the illegal market, being the most potent and fastest acting analogue. It is typically available in the form of tablets or as a white crystalline powder, named as “crystal meth,” “glass,” or “ice” [4, 5, 6], acting as a sympathomimetic agent and exerting adverse effects across multiple biological systems [4, 5, 6]. Due to its asymmetric nature, MA exists as two enantiomers, (*S*)‐(+)‐MA **(1)** and (*R*)‐(−)‐MA **(2)**, each exhibiting distinct and clinically relevant biological activities (Figure 1) [7]. The (*S*)‐(+)‐MA **(1)** enantiomer remains legally prescribed for the treatment of attention deficit and hyperactivity disorders and as an appetite suppressant [6, 7, 8]., whereas the (*R*)‐(−)‐MA **(2)** enantiomer is widely incorporated into over‐the‐counter nasal decongestants formulations [9, 10, 11].

Although significant progress has been made in this field and considerable effort has been devoted to elucidating the biological activity of each MA enantiomer, their effects remain incompletely understood. Accordingly, this integrative review critically examines the current scientific literature on MA enantiomers, with particular emphasis on the biological and chemical distinctions between (*S*)‐(+)‐MA **(1)** and (*R*)‐(−)‐MA **(2)**. The discussion highlights their structural, pharmacological, toxicological, and synthetic differences, providing a comprehensive overview of their respective roles within biological systems.

## 2 Biological and Pharmacological Perspectives

Methamphetamine (MA) abuse is associated with serious health implications [12, 13, 14], with limited treatment options. The most promising strategies currently involve psychological interventions, particularly contingency management approaches [15]. From a biological perspective, the following sections discuss each aspect of the two MA enantiomers, beginning with the routes of administration and absorption, followed by biodistribution and pharmacodynamics, metabolism, and finally excretion.

## 3 Distribution and Pharmacodynamics

### 3.1 Central Nervous System (CNS)

The chemical structure of methamphetamine (MA) presents an important aspect that facilitates its cross through the blood–brain barrier (BBB). It can be attributed to the presence of an *N*‐methyl group in its structure [7], which results in greater lipid solubility than amphetamine (AT) [16], leading to a more rapid and pronounced onset of effects within the CNS [7, 17].

After reaching the CNS, MA acts as a substrate‐type releaser across multiple neurotransmitter systems, such as serotonin (SERT) and glutamate, but notably dopamine (DA) and norepinephrine (NE) [18]. MA binds to transporter proteins, being internalized into nerve terminals, thereby disrupting the normal activity of these transporters [19]. With respect to DA, the MA‐induced increase in dopaminergic signaling is mainly associated with alterations in the dopamine transporter (DAT) and the vesicular monoamine transporter‐2 (VMAT‐2) [20, 21].

With respect to NE, MA exerts similar actions through interactions with the plasma norepinephrine transporter (NET) and VMAT‐2, engaging mechanisms analogous to those described for DA. MA competitively inhibits NET [22], promotes NE efflux into the cytoplasm [23], and disrupts the electrochemical gradient required for proper NE storage [24, 25]. Beyond these main effects, NE plays a fundamental role in the regulation and release of DA, increases locomotor activity and directly stimulates motivated behaviors [26].

According to a work published recently by Elder and co‐workers, the enantiomers (*S*)‐(+)‐MA **(1)** and (*R*)‐(−)‐MA **(2)** exhibit distinct activities in the CNS, mainly in terms of overall potency and monoaminergic selectivity. The authors performed in vitro studies aiming to the evaluation of the pharmacodynamic profiles of each enantiomer, assessing both the potential for monoamine release via DAT, NET, and SERT. Comparative analysis indicated that (*S*)‐(+)‐MA **(1)** displays higher affinity and potency for both dopamine release and reuptake inhibition (DAT), with approximately 10 times greater selectivity relative to (*R*)‐(−)‐MA **(2)**, reflecting its predominantly dopaminergic activity. In contrast, (*R*)‐(−)‐MA **(2)** exhibits lower overall affinity and pharmacological potency, consistent with its selective activity at the norepinephrine (NET) inhibitor and its predominant role in noradrenergic modulation. Both enantiomers demonstrate low affinity for SERT; however, (*S*)‐(+)‐MA **(1)** remains more active than (*R*)‐(−)‐MA **(2)** in this context [27, 28].

### 3.2 Psychomotor Effects

The disparities in monoaminergic selectivity and potency between MA enantiomers are fundamental to their divergent physiological and behavioral profiles. Preclinical investigations in rodents' models provided evidence that the pronounced dopaminergic selectivity of (*S*)‐(+)‐MA **(1)** underlies its sympathomimetic action, observed in psychomotor effects, which are greater than those triggered by (*R*)‐(−)‐MA **(2)** [29, 30, 31]. The work published by Xue and co‐workers pointed out that the administration of (*S*)‐(+)‐MA **(1)** displays a potent, linear, and dose‐dependent increase in locomotion activity at low doses ranging from 0.32 to 3 mg/kg [30]. A comparable dose‐dependent pattern was observed in the manifestation of stereotypic behaviors, characterized by intense repetitive head and paws movements, and occasionally by biting, licking, or sniffing, in the absence of ambulation. In contrast, administration of (*R*)‐(−)‐MA **(2)** at equivalent doses fails to evoke comparable hyperlocomotive or stereotypic responses.

At higher doses, such as 3.2, 5.0, and 10.0 mg/kg [29, 30, 31], the hyperlocomotion response induced by (*S*)‐(+)‐MA **(1)** is attenuated and supplanted by stereotyped behaviors. At these same doses, administration of (*R*)‐(−)‐MA **(2)** produces a significant increase in both locomotor activity and stereotypies, effects that are not detected at lower dose ranges.

The dopaminergic selectivity of (*S*)‐(+)‐MA **(1)** increases the dopamine action in the *nucleus accumbens* (NAc), a fundamental structure involved in motor functions, thereby accounting for the dose‐dependent hyperlocomotion observed at lower doses. At higher doses, intensified dopaminergic effects manifest as prominent stereotypic behavior.

Conversely, due to the preferential noradrenergic activity of (*R*)‐(−)‐MA **(2)**, hyperlocomotor effects are not produced at low doses. However, given the modulatory influence of NE on DA [32], greater noradrenergic stimulation at higher doses may have contributed to the observed increases in locomotor activity and stereotypy.

Furthermore, recent investigations performed by Wang and co‐workers employed *Caenorhabditis elegans* models, at doses of 1.25, 2.50, 5.00, and 10.00 mM, to further corroborate these enantiomer‐induced locomotor activities. Similar to rodents studies, (*S*)‐(+)‐MA **(1)** elicited more potent acute motor responses, whereas (*R*)‐(−)‐MA **(2)** produced comparatively attenuated effects on locomotor activity [33].

### 3.3 Cardiovascular Effects

Cardiovascular complications represent one of the principal causes of morbidity and mortality associated with MA abuse [34]. A work published by Fowler and co‐workers described Positron Emission Tomography (PET) studies performed on nonhuman primates, which demonstrated that both MA enantiomers are rapidly taken up and subsequently cleared from cardiac tissue [35]. Consistent with these findings, a subsequent human study published by Volkow and co‐workers, following intravenous administration of (*S*)‐(+)‐MA **(1)**, has revealed rapidly myocardial uptake, characterized by low peak concentration and an intermediate clearance of approximately 16 min [34].

The pharmacodynamic properties of MA enantiomers, particularly in relation to cardiovascular parameters, were investigated in a clinical study by Mendelson and co‐workers [7]. The results indicated that the (*S*)‐(+)‐MA **(1)** produced greater increases in heart rate and systolic blood pressure than the (*R*)‐(−)‐MA **(2)**. Administration of (*S*)‐(+)‐MA **(1)** at 0.5‐mg/kg elevated systolic blood pressure by approximately 33–34 mmHg, whereas (*R*)‐(−)‐MA **(2)** caused a moderate increase of 19 mmHg. Subjectively, effects such as euphoria and intoxication were longer lasting with (*S*)‐(+)‐MA **(1)** (around 6 h) than with (*R*)‐(−)‐MA **(2)** (around 3 h). The racemic mixture exhibited a response profile similar to (*S*)‐(+)‐MA **(1)**, indicating that (*R*)‐(−)‐MA **(2)** does not attenuate the cardiovascular effects. A precedent clinical study from 1995 confirmed that administration of (*S*)‐(+)‐MA **(1)** leads to significant increases in systolic and diastolic blood pressure, heart rate, and the frequency‐pressure product, a consequence of elevated systolic blood pressure [36].

A subsequent study by Mendelson and colleagues [9] examined the pharmacology of (*R*)‐(−)‐MA **(2)**, the isomer associated with lower abuse potential, when administered as a nasal decongestant. At low doses, (*R*)‐(−)‐MA **(2)** marketed under the trade name *Vicks*, produced minimal cardiovascular effects in healthy people, even at doses exceeding the recommended therapeutic levels. Over time, only minor changes in blood pressure and heart rate were observed, all of which were classified as clinically insignificant.

### 3.4 Respiratory Effects

The respiratory system represents an important pharmacological target affected by the systemic actions of MA. In nonhuman primates evaluated by PET, both (*S*)‐(+)‐MA **(1)** and (*R*)‐(−)‐MA **(2)** exhibited similar pharmacokinetics profiles, characterized by rapid pulmonary uptake and clearance [35]. Comparable findings have been reported in humans, where intravenous administration of (*S*)‐(+)‐MA **(1)** resulted in rapid lung uptake, within approximately 55 s, reaching high concentration peaks followed by rapid clearance [34].

In rodents' studies, (*S*)‐(+)‐MA **(1)** produced respiratory stimulatory effects, inducing a significant dose‐dependent increase in volumetric ventilation (MVb, mL/min) at doses of 1, 3, and 10 mg/kg. In contrast, administration of 1‐ and 3‐mg/kg doses of (*R*)‐(−)‐MA **(2)** induced respiratory depression, reducing MVb by approximately 43% and 49% from baseline, respectively. At a 10‐mg/kg dose, (*R*)‐(−)‐MA **(2)** did not produce significant effects at any time point. When the dose was increased to 30 mg/kg, (*R*)‐(−)‐MA **(2)** elicited respiratory stimulation compared to that observed with 3 mg/kg of (*S*)‐(+)‐MA **(1)**. Therefore, both (*S*)‐(+)‐MA **(1)** and (*R*)‐(−)‐MA **(2)** display dose‐dependent respiratory effects; however, these effects are opposite for most of the dosages, and similar dose‐dependent patterns were also observed in respiratory rate (breaths/min) [27].

Human studies depicted by Mendelson and co‐workers further supported these findings. Manual assessment of breathing frequency following intravenous administration of MA enantiomers at 0.25‐ and 0.50‐mg/kg doses demonstrated that (*S*)‐(+)‐MA **(1)** exerted the greatest stimulatory effect on respiratory rate [8]. In contrast, (*R*)‐(−)‐MA **(2)**, which has been widely used in vasoconstrictive nasal decongestant formulations, produced no clinically significant effects in respiratory rate when administered by inhalation, even at doses two to four times higher than those recommended therapeutically [9].

## 4 Metabolism

Methamphetamine is primarily metabolized in the liver, which shows the highest concentrations of MA following administration in humans. Similar findings have been reported in baboons. Despite this high hepatic uptake, MA clearance is slow, taking more than 75 min in humans [34, 35].

**FIGURE 2:**
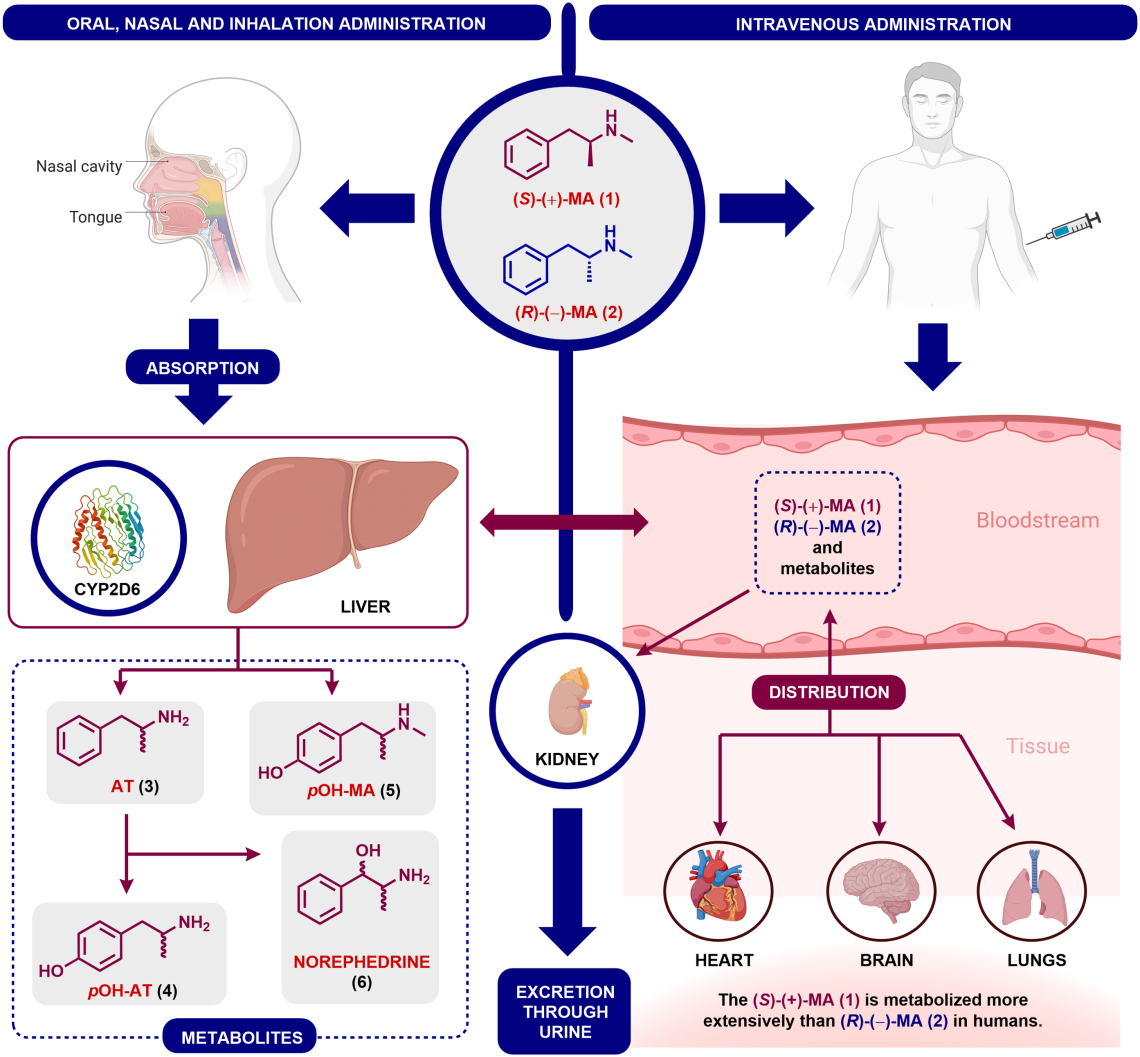
MA metabolism by the CYP2D6 isoenzyme in human (Image created using Biorender.com).

Metabolism of MA occurs mainly via CYP2D6 isoenzyme of the cytochrome P450 system. The main phase I pathways are *N*‐demethylation, producing AT **(3)**, and aromatic hydroxylation, yielding *p*‐hydroxyMA (*p*OH‐MA, **5**) [37]. These metabolites may undergo Phase II conjugation with glucuronic acid or sulfate, facilitating excretion. The AT formed can also be transformed into *p*‐hydroxyamphetamine (*p*OH‐AT, **4**) [38] or norephedrine **(6)** through *beta*‐hydroxylation [38] (Figure 2).

Regarding the stereoselectivity on liver, a pivotal study conducted by Sevcika and co‐workers evaluated the metabolism of both enantiomers of MA from human urine samples. This work clearly indicated that each enantiomer follows a different metabolic pathway, with (*S*)‐(+)‐MA **(1)** metabolized more extensively than (*R*)‐(−)‐MA **(2)** [39, 40].

A further analysis on the different metabolism of MA enantiomers was studies by Li and co‐workers. The authors described that the formation of (*S*)‐(+)‐AT from (*S*)‐(+)‐MA **(1)** is significantly greater than that of (*R*)‐(+)‐AT from (*R*)‐(−)‐MA **(2)** [41], though this difference decreases over time, indicating that (*S*)‐(+)‐MA is metabolized more completely and rapidly [42]. Following intravenous administration, the AUC of (*S*)‐(+)‐MA is higher than that of its R enantiomer, especially after oral administration, indicating stronger first‐pass metabolism [42, 43]. Conversely, (*R*)‐(−)‐MA **(2)** is more likely excreted unchanged.

## 5 Excretion

Methamphetamine is rapidly absorbed and reaches high concentrations in kidneys [34, 35], reflecting its predominant urinary excretion. Between 37% and 54% of MA is eliminated unchanged in urine [44, 45]. The half‐life (t_½_) and clearance of MA vary by route of administration. Intranasal and inhalation (smoking) routes yield a t_½_ of around 10.7 h and bioavailability approximately from 67% to 90% [46, 47]. Intravenous administration results in a t_½_ of approximately 11.4 h [48], and bioavailability of 100%. Oral administration, on the other hand, presents a t_½_ average of 9.3 ± 3.7 h at low doses (10 mg/day) and 11.1 ± 7.2 h at higher doses (20 mg/day) [49], and bioavailability of approximately 67% [50, 51].

Methamphetamine is also excreted via breast milk. Among women who smoked MA, the t_½_ in breast milk ranged from 11.3 to 30.3 h, with complete elimination occurring after approximately 100 h. Furthermore, for intravenous administration of lactating mothers, t_½_ values up to 43 h have been reported, indicating a potential risk to nursing infants [44].

Stereochemical differences significantly influence MA pharmacokinetics. Mendelson and co‐workers described that the t½ of (*S*)‐(+)‐MA **(1)** ranges from 10 to 11 h, whereas (*R*)‐(−)‐MA **(2)** exhibits a slightly longer t½ of 13–15 h [7], which explains their clearance difference. This may lead to plasma accumulation of the *R* enantiomer during repeated racemic dosing.

## 6 Analytical Methods for the Identification and Quantification of Enantiomers in Biological Matrices

Analytical discrimination of MA enantiomers plays a central role in pharmacological, toxicological, and forensic studies. Although it remains a challenge, requiring the development of methods that provide high sensitivity and robust enantiomeric discrimination. Several analytical approaches have been applied to many biological matrices, including blood, urine, hair, and saliva.

Urine remains the most extensively analyzed biological matrix for identification and quantification of MA enantiomers and their corresponding metabolites. It is particularly valuable for distinguishing illicit and therapeutic sources employed for enantiomer‐based medications or their precursors [52].

Over the recent years, Helander and co‐workers [53], through liquid chromatography–tandem mass spectrometry (LC–MS/MS), reported that low concentrations of MA in urine samples containing high concentrations of amphetamine (AM) do not necessarily highlight the application of multiple drugs but may instead be associated with the *N*‐methylation of amphetamine. In contrast, employing the same analytical method with a chiral column (Chirobiotic V2), urine samples collected from individuals suspected of MA abuse exhibited a predominance of the (*S*)‐(+)‐MA **(1)** and (*S*)‐(+)‐AT enantiomers, whereas no samples tested positive exclusively for (*R*)‐(−)‐MA **(2)** [54].

In the same context, the 1991 Technical Guideline issued by the US Department of Health and Human Services (HHS) established that the presence of more than 20% (*S*)‐(+)‐MA **(1)** in samples such as urine is indicative of exposure to a source other than an over‐the‐counter product [55, 56].

Enantiodiscrimination of methamphetamine (MA) and amphetamine (AT) was achieved using gas chromatography–mass spectrometry (GC–MS) in urine samples collected following the controlled administration of Vicks VapoInhaler. The results revealed the exclusive detection of (*R*)‐(−)‐MA **(2)** in the samples. Two urine samples exhibited concentrations below the ≥ 250‐μg/L threshold, which is the reference cutoff value established for federal workplace drug testing program [10]. The controlled administration of Vicks VapoInhaler was also evaluated by using an enantioselective LC–ESI–MS/MS method following derivatization with Marfey's reagent. In human oral fluid samples, 98% contained more than 20% (*S*)‐(+)‐MA **(1)** enantiomer [56]. In a separate study, the standardization and validation of an enantioselective method demonstrated adequate sensitivity and selectivity for the discrimination and quantification of MA and AT enantiomers in oral fluid and plasma [57].

Similarly, Esposito and co‐workers [55] evaluated commercial nasal inhalers using GC–MS after chiral derivatization with *N*‐trifluoroacetyl‐*L*‐prolyl chloride (L‐TPC). The results indicated less than 1% of the (*S*)‐(+)‐MA **(1)** enantiomer. The same chiral derivatization reagent had been previously employed in combination with GC–MS for the simultaneous determination of MA and AT enantiomers in urine [58].

Additionally, the discrimination of enantiomers in urine has also been demonstrated using immunoassay‐based approaches. An immunoaffinity chromatography (IAC) column coupled with LC–MS was developed using anti‐(*S*)‐(+)‐MA **(1)** mouse monoclonal antibodies immobilized on a silica gel support. This method provides high immunological specificity and reduces false‐positive results associated with the presence of (*R*)‐(−)‐MA **(2)** in urine samples [59]. Alternative approaches such as gas chromatography coupled with a nitrogen–phosphorus detector (GC–NPD) have also proven effective investigating MA derived from therapeutic use in urine samples collected during antidoping testing [60].

Regarding enantioselective analysis in blood samples, LC–MS/MS has been employed not only for the chromatographic separation of MA and AT enantiomers but also to investigate whether the consumed MA was produced through a racemic or stereoselective synthesis pathway in some regions of Germany [61]. Using whole blood samples, Rasmussen and co‐workers [62] achieved chiral separation and quantification of MA enantiomers through derivatization with *R*‐MTPCl and analysis using an HP‐5MS column coupled to GC–EI–MS. Additionally, using GC–NICI–MS, MA enantiomers were compared in plasma samples obtained from screening, intoxication, and drug‐impaired driving cases in Belgium.

Bickel and co‐workers [63] reported the development of an analytical method for hair samples, enabling the simultaneous identification of (*R*/*S*)‐enantiomers of various substances, including MA, in a single LC–MS/MS analysis. The method was based on a derivatization procedure with dansyl chloride and enantiodiscrimination using a chiral stationary phase. Similarly, the same research group applied this method for the identification of (*R*/*S*)‐enantiomers in postmortem hair samples [64]. Additionally, Xiang and co‐workers used chiral LC–MS/MS to quantify both enantiomers in hair samples from 13 female former MA abusers undergoing remission. The results indicated long‐term accumulation with a predominance of (*S*)‐(+)‐MA **(1)** [50]. The same finding was reported by Martins and co‐workers [65].

In another study, the same method was applied to evaluate the amphetamine‐to‐methamphetamine ratio. The authors reported an increase in this ratio correlated with the duration of MA abuse, potentially providing insights into retrospective drug exposure and patterns of use [66]. The influence of cosmetic hair bleaching treatments on MA enantiomeric ratios was also investigated. Although untreated hair samples tested positive for both enantiomers, samples subjected to bleaching showed 39% reduction in concentrations. Gas chromatography–mass spectrometry in negative ion chemical ionization mode (GC/MS‐NICI) was used to identify the isomers [67].

The enantiodiscrimination and quantification of MA in biological matrices have been achieved using various analytical methods, including derivatization with Marfey's reagent [56, 68], with achiral [69] fluorescent [70] agents, fluorescence detection [71], chiral stationary phase coupled with tandem mass spectrometry (CSP‐LC–MS/MS) [72], liquid chromatography coupled with electrospray ionization mass spectrometry (HPLC‐ESI‐MS) [73], GC/MS‐NICI [74, 65, 75, 76], high‐performance liquid chromatography with (HPLC) fluorescence detection [77, 78], and capillary electrophoresis [79, 80, 81, 82].

### 6.1 Synthetic Procedures Towards Methamphetamine

A wide variety of synthetic routes exist for the obtention of MA, each using distinct precursors and reagents, and producing characteristic contaminants, sub‐products, and impurities [83]. Landmark contributions in this matter were published by Nagai [84] and Ogata [85], from which MA was achieved from *l*‐ephedrine (**7**) or 1‐phenyl‐2‐propanone (P2P, **8**), respectively.

Other important synthetic routes developed over the 20th and 21st centuries illustrate the historical and chemical diversity of methods that converge on P2P (**8**) as a central intermediate, leading to racemic MA [86, 87]. Despite the contribution of these works, it is important to mention that enantiospecific syntheses were still incipient at this time, which led to major contributions that will be discussed in the following sessions.

## 7 Synthetic Approaches to Enantiopure Methamphetamine

Like other chiral compounds, the obtention of enantiopure MA requires specific and well‐designed synthetic strategies. Without stereochemical control, syntheses typically yield a racemate, and as discussed earlier, the two enantiomers exhibit distinct biological effects. Understanding methods that produce enantiopure (*S*)‐(+)‐MA **(1)** or (*R*)‐(−)‐MA **(2)** is therefore essential.

Although many studies describe nonstereoselective syntheses followed by enantiomeric resolution [88], this review focuses instead on enantioselective approaches, either using inherited chirality from enantiopure substrates or asymmetric induction through enantioselective catalysis. The following sections address methods based on ephedrine **(7)**, chiral heterocyclic intermediates, and other general enantioselective routes.

### 7.1 Using Ephedrine as Substrate

**SCHEME 1:**
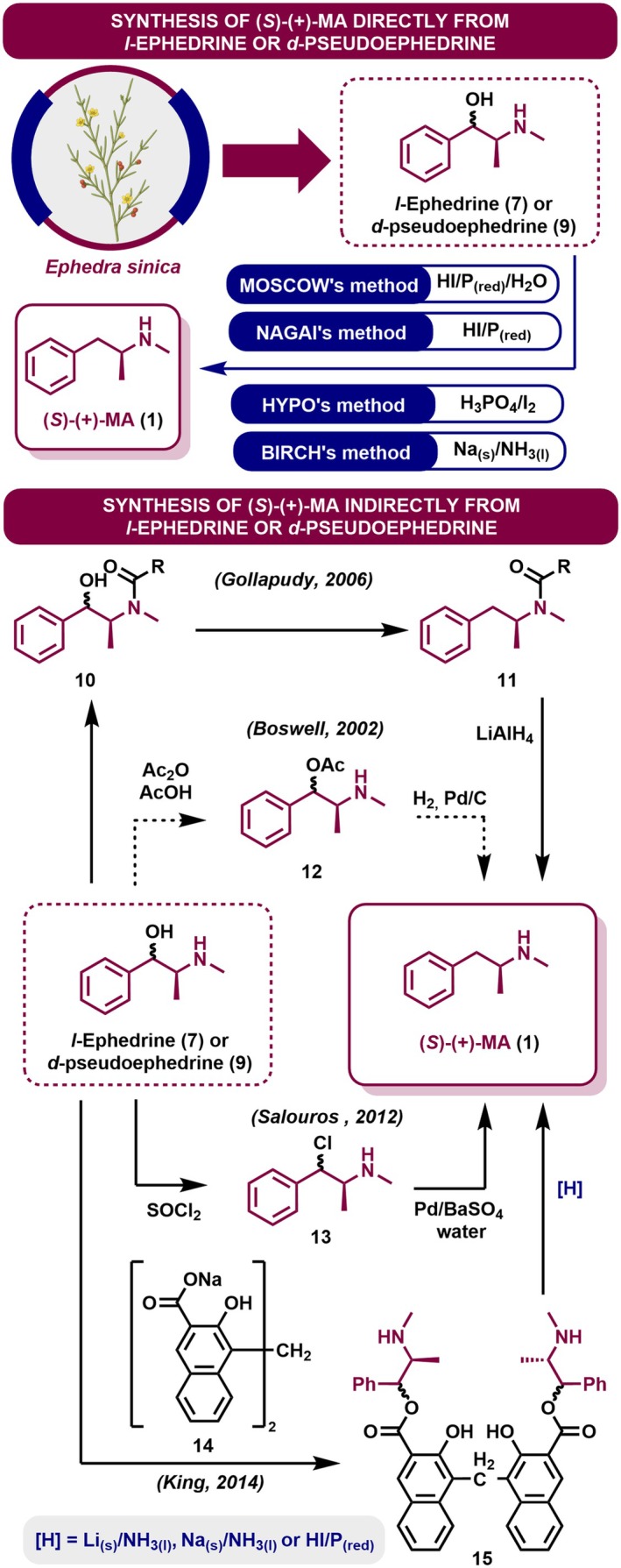
Synthetic approaches towards (*S*)‐(+)‐MA **(1)**, using ephedrine **(7** or **9)** as starting material.

Two important enantiopure precursors commonly used for the obtention of (*S*)‐(+)‐MA **(1)** are *l*‐ephedrine **(7)** and *d‐*pseudoephedrine **(9)**. Both compounds are natural alkaloids extracted from the *Ephedra* plant [89]. Several well‐stablished methods, such as the previously mentioned Nagai's method, also Hypo, Moscow and Birch synthetic routes (Scheme 1) [84, 90], enable conversion to (*S*)‐(+)‐MA **(1)** in a single step. Due to their simplicity and the accessibility of reagents (e.g., lithium from car batteries and red phosphorous from matchboxes), these methods are frequently adopted in clandestine laboratories [91].

Structural modifications, reported by Gollapudy and co‐workers, at the *N*‐methyl portion of *l*‐ephedrine **(7)** have also been reported, through which *N‐*acetylation yielded intermediate **10**, followed by further two steps leading to enantiopure (*S*)‐(+)‐MA **(1)** [92]. This method uses the inheritance of the enantiospecificity observed on the natural compound itself, facilitating the obtention of enantiopure samples.

Salouros and co‐workers revisited the Emde and Nagai methods, both of which functionalize *l*‐ephedrine **(7)** using iodine and phosphorus (Scheme 1). The Emde method proceeds via substitution of the hydroxyl group with chlorine, followed by reduction of intermediate **13** to produce (*S*)‐(+)‐MA **(1)** [93].

Later, King and co‐workers [94] disclosed a patent describing MA synthesis from *l*‐ephedrine **(7)** or *d*‐pseudoephedrine **(9)** derivatives via either Birch's reduction or the hydriodic acid/red phosphorous route (Scheme 1). The authors showed that pamoate salts **(14)** lead to the formation of an ester intermediate **(15)** which, under reductive conditions, retain the stereochemistry of (*S*)‐(+)‐MA **(1)**.

### 7.2 Using Heterocyclic Substrates

**SCHEME 2:**
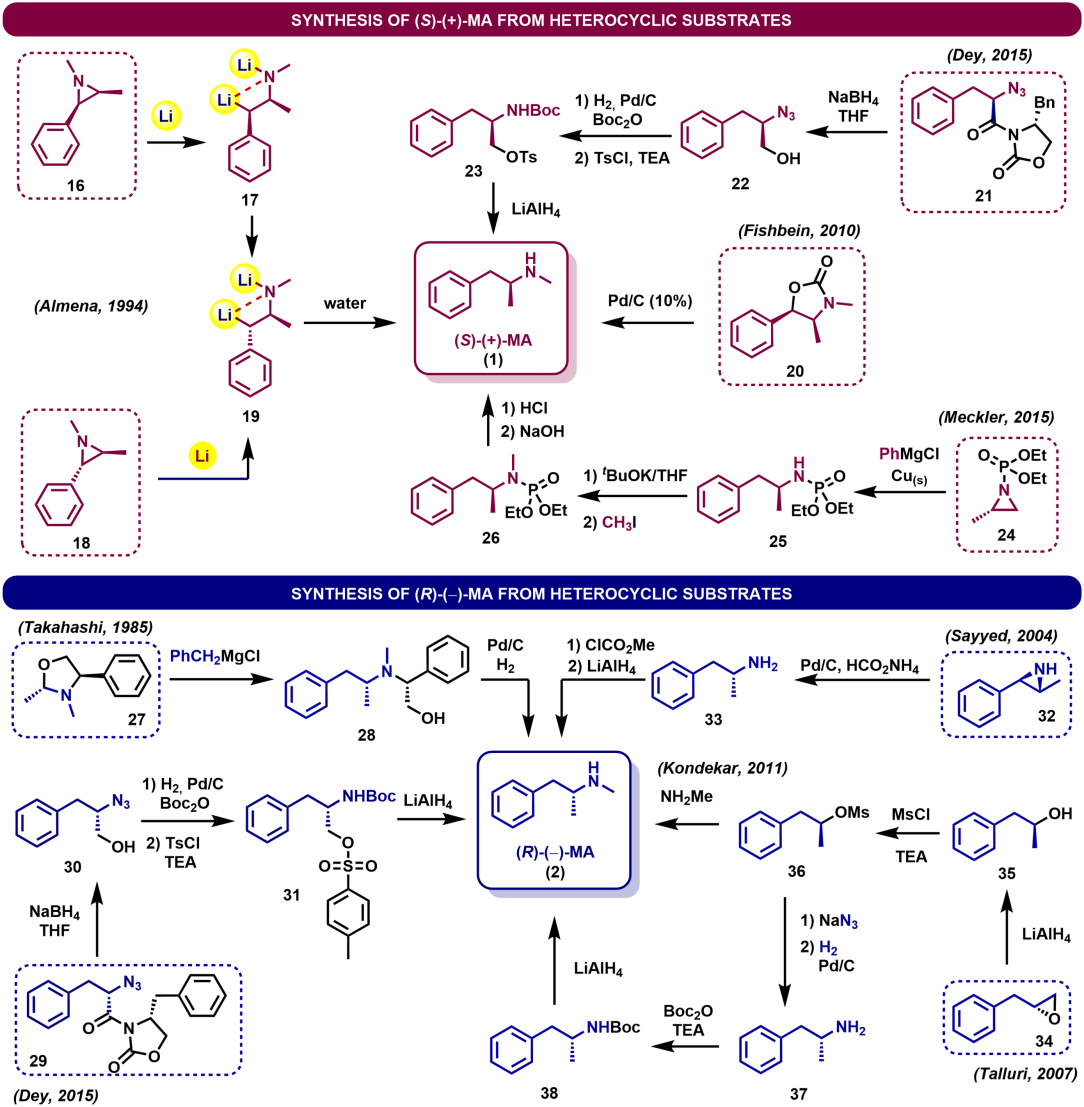
Synthetical approaches towards (*S*)‐(+)‐MA **(1)** and (*R*)‐(−)‐MA **(2)**, using heterocyclic substrates as starting materials.

The enantioselective synthesis of MA from heterocyclic precursors has been explored through several innovative approaches. These strategies highlight the versatility of nitrogen‐containing heterocycles as starting materials and underscore the central role of stereoselective transformations in psychostimulant chemistry (Scheme 2).

For (*S*)‐(+)‐MA **(1)**, Almena demonstrated that the aziridines **16** and **18** could undergo nucleophilic ring‐opening with lithium reagents to introduce the phenyl group in a stereoselective manner (Scheme 2) [95]. Later, Dey employed heteroaryl azides (e.g., compound **21**) as substrates for the obtention of (*S*)‐(+)‐MA **(1)**. In this method, the substrate is reduced with sodium borohydride towards **22**, and after protecting group manipulations and tosylation, intermediate **23** can be achieved and further reduced and deprotected to the target amine [96]. Meckler reported a copper‐catalyzed Grignard addition to a phosphorus‐containing aziridine **24**, forming intermediate **25** with the benzylic framework [97]. Additionally, Fishbein used an oxazolidinone substrate **20** under Pd/C catalysis to deliver the desired enantiomer (Scheme 2) [98]. Collectively, these pathways demonstrate that aziridine, oxazolidinone, and related heterocycles provide robust scaffolds for accessing enantiopure (*S*)‐(+)‐MA **(1)**.

The enantioselective synthesis of (*R*)‐(−)‐MA **(2)** has also been explored. Takahashi pioneered this field by utilizing chiral oxazolidines; the addition of benzylmagnesium chloride to the substrate **27** furnished intermediate **28**, which subsequently underwent hydrogenolysis to yield the desired (*R*)‐(−)‐MA **(2)** [99]. In a similar approach, Dey employed reductive cleavage on a chiral *N*‐acylated cyclic carbamide **(29)** to generate the azido‐alcohol **30**, followed by a catalytic hydrogenation to the corresponding amine, and subsequent *N*‐protective tosylation to form **31**, and finally a LiAlH_4_ reduction yielded the target enantiomer [96]. Additionally, Sayyed achieved an efficient route through catalytic hydrogen‐transfer opening of the aziridine‐derived substrate **32** to deliver amine **33**, which was subsequently converted to (*R*)‐(−)‐MA **(2)** via an *N*‐monomethylation process [100]. Talluri reported a route involving the conversion of epoxide **34** to mesylate **36**, which led to (*R*)‐(−)‐MA **(2)** through a multistep pathway [101]. However, Kondekar developed a flexible protocol in which mesylate **36** could undergo direct nucleophilic displacement with methylamine to selectively afford the desired (*R*)‐(−)‐MA **(2)** [102].

### 7.3 Other Synthetic Strategies

Beyond the well‐established routes starting from *l*‐ephedrine **(7)** derivatives and the more recent heterocyclic substrates strategies, several alternative enantioselective approaches to MA have also been explored. Though less conventional, these methods illustrate the breadth of synthetic logic applicable to access this chiral stimulant.

**SCHEME 3:**
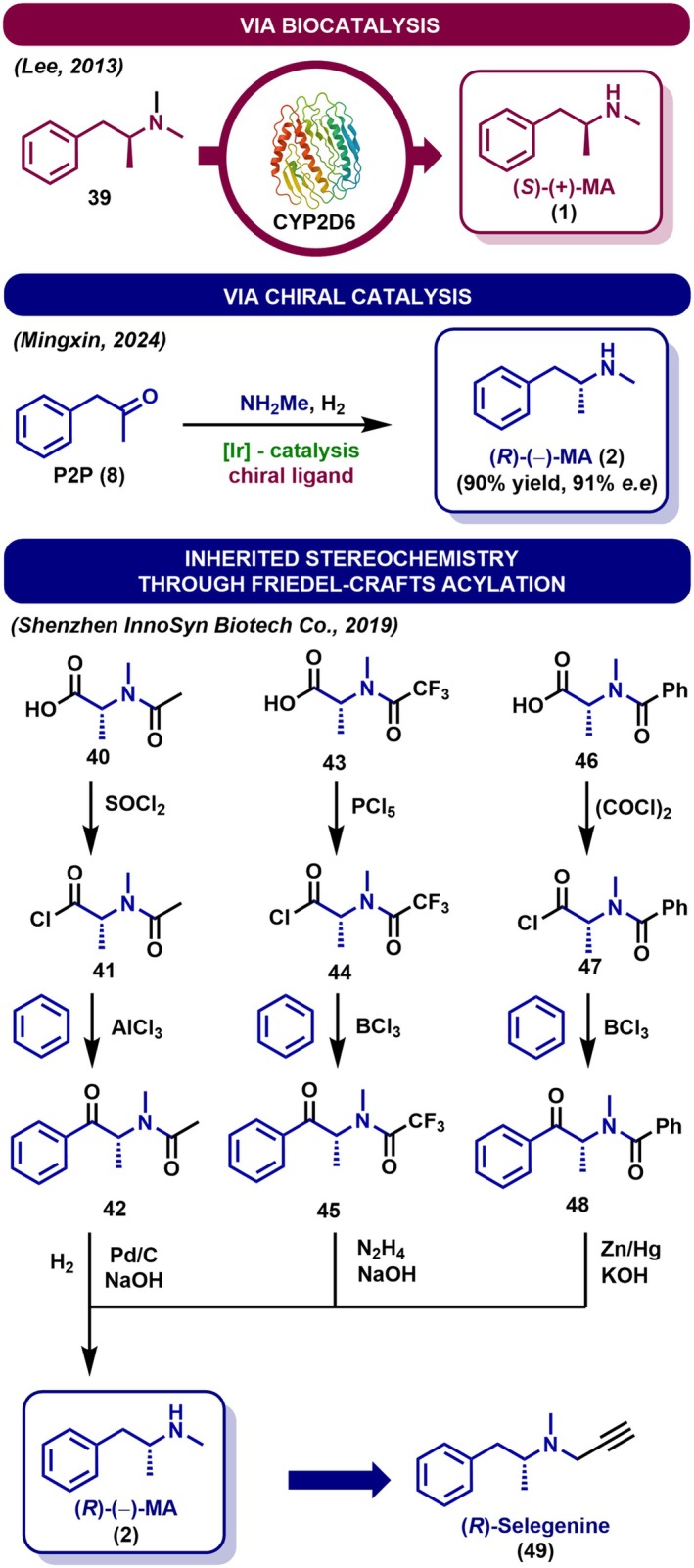
Contemporaneous enantioselective synthetic approaches towards (*S*)‐(+)‐MA **(1)** and (*R*)‐(−)‐MA **(2)** (Image created using Biorender.com).

Lee and co‐workers described a biological conversion of *N*,*N*‐dimethylamphetamine **(39)** into MA [103], demonstrating that each enantiomer follows a distinct metabolic pathway. The (*S*) enantiomer underwent *N*‐monodemethylation, yielding (*S*)‐(+)‐MA **(1)** (Scheme 3). Later, in 2019, Shenzhen InnoSyn Biotech Co. reported a patent outlining three synthetic routes to (*R*)‐selegiline **(49)** through functionalization of (*R*)‐(−)‐MA **(2)** [104], which was obtained via Friedel–Crafts reactions from the corresponding carboxylic acids **40**, **43**, and **46** (Scheme 3). More recently, Mingxin and co‐workers published a patent [105], introducing an iridium‐catalyzed asymmetric reductive amination towards (*R*)‐(−)‐MA **(2)**. For this matter, primary amines and P2P **(8)** were employed as substrates, under catalysis by an iridium catalyst, generated in situ in the presence of a chiral phosphoramidite ligand (Scheme 3). Collectively, these approaches highlight the chemical creativity driving asymmetric MA synthesis and provide a framework for future advances in the enantioselective preparation of pharmacologically relevant compounds.

## 8 Purification Procedures Towards the Obtention of Pure Enantiomers

**FIGURE 3:**
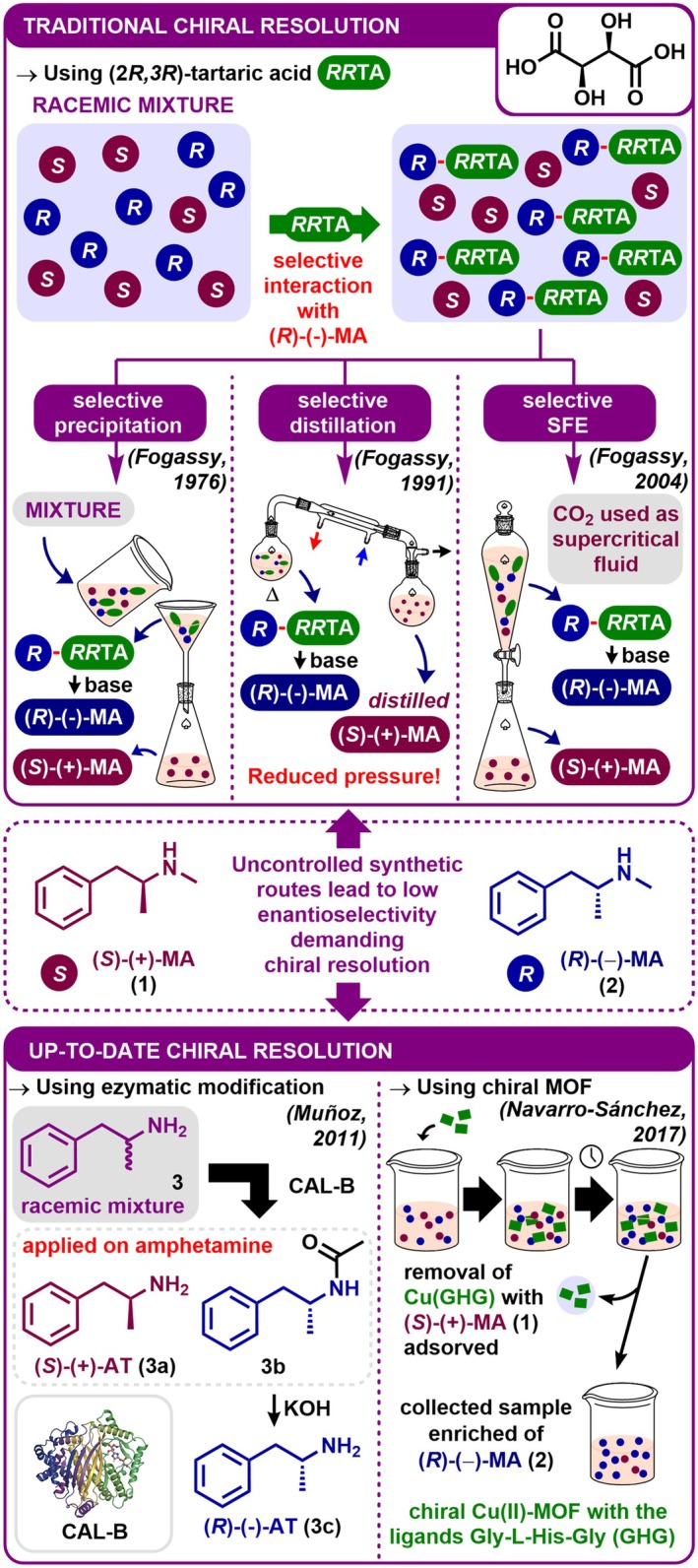
Chiral resolution of amphetamine derivative mixtures.

Although this work is focused on enantioselective syntheses of (*S*)‐(+)‐MA **(1)** or (*R*)‐(−)‐MA **(2)**, it is important to briefly address the strategies commonly employed for the purification and isolation of these enantiomers. Synthetic methods that lack enantioselectivity, such as the Leuckart synthetic approach, yield racemic mistures [86, 87]. Conversely, even enantioselective routes with modest enantiomeric excess may require an additional chiral resolution step to enhance the enantiopurity of the isolated product, particularly when the synthesis is intended for medicinal applications. Traditionally, the resolution of racemic methamphetamine or its synthetic precursors relies on classical diastereomeric crystallization using chiral resolving agents, such as (2*R*,3*R*)‐tartaric acid or (2*S*,3*S*)‐tartaric acid [106, 107, 108, 109, 110], followed by subsequent conversion into their respective hydrochloride salts. In these methods, a transient diastereoisomeric mixture is formed, which can be separated based on differences in physical–chemical properties, typically solubility, polarity, or boiling point. Over the past decades, the Fogassy research group has made valuable contributions to this area, including separation via selective crystallization of the favorable Lewis adduct between (*S*)‐(+)‐MA **(1)** and (2*S*,3*S*)‐tartaric acid (*SS*TA), or between (*R*)‐(−)‐MA **(2)** and (2*R*,3*R*)‐tartaric acid (*RR*TA) [106, 107, 108, 109, 110]. According to their description of the Pope‐Peachey Method, when a racemic mixture of (*S*)‐(+)‐MA **(1)** and (*R*)‐(−)‐MA **(2)** is treated with *RR*TA and aqueous HCl, both (*S*)‐(+)‐MA/*RR*TA and (*R*)‐(−)‐MA/*RR*TA solid Lewis adducts are formed, along with the corresponding soluble hydrochloride salts [108]. However, the equilibrium tends to favor the formation of (*R*)‐(−)‐MA/*RR*TA and (*S*)‐(+)‐MAH^+^Cl^−^ over time. The group also described successful resolution using reduced‐pressure distillation [107] or supercritical fluid extraction (SFE) [110] with supercritical carbon dioxide (Figure 3).

While effective, these methods often require multiple recrystallization or redistillation steps to achieve high enantiomeric purity. To circumvent these limitations, modern separation strategies have increasingly focused on innovative chiral resolution approaches [111, 112, 113, 114]. For instance, Muñoz and co‐workers described a biological enantiospecific recognition process for the racemic mixture of amphetamine (**AT**, **3**), using the enzyme CAL‐B [111]. In this process, only the (*R*)‐(−)‐MA (**3c**) is acetylated to compound **3b**.

Thus, (*S*)‐(+)‐MA (**3a**) can get isolated, whereas (*R*)‐(−)‐MA (**3c**) can be obtained from **3b** after a basic hydrolysis (Figure 3). Another significant contribution to this area was reported by Navarro‐Sánchez and co‐workers, who described the design of an advanced homochiral three‐dimensional metal–organic framework (MOFs) constructed from naturally occurring chiral oligopeptide, capable of high‐efficiency chiral recognition [112].

Specifically, they developed a peptide‐based MOF Cu (GHG), which utilizes the tripeptide glycyl‐L‐histidylglycine to decorate its internal 1D channels with a dense array of versatile functional groups, including imidazole, amide, and carboxylate moieties. Computational simulations and solid‐phase extraction experiments demonstrated that these frameworks exhibit remarkable stereoselectivity, preferentially capturing (*S*)‐(+)‐MA **(1)** over its counterpart through optimized noncovalent interactions and hydrogen bonding within the chiral pockets. Consequently, the incorporation of such advanced crystalline matrices represents a prominent trend to complement enantioselective synthesis, enabling rapid and precise isolation of pure amphetamine‐type stimulants in forensic, clinical, and pharmaceutical applications.

### 8.1 Methamphetamine‐Derivatived Medicinal Drugs

Despite the well‐documented and often detrimental effects associated with addictive compounds, such as MA [115, 116, 117], it is important to recognize that many of these substances, as well as their derivatives and metabolites, possess significant pharmacological potential [118, 119].

**FIGURE 4:**
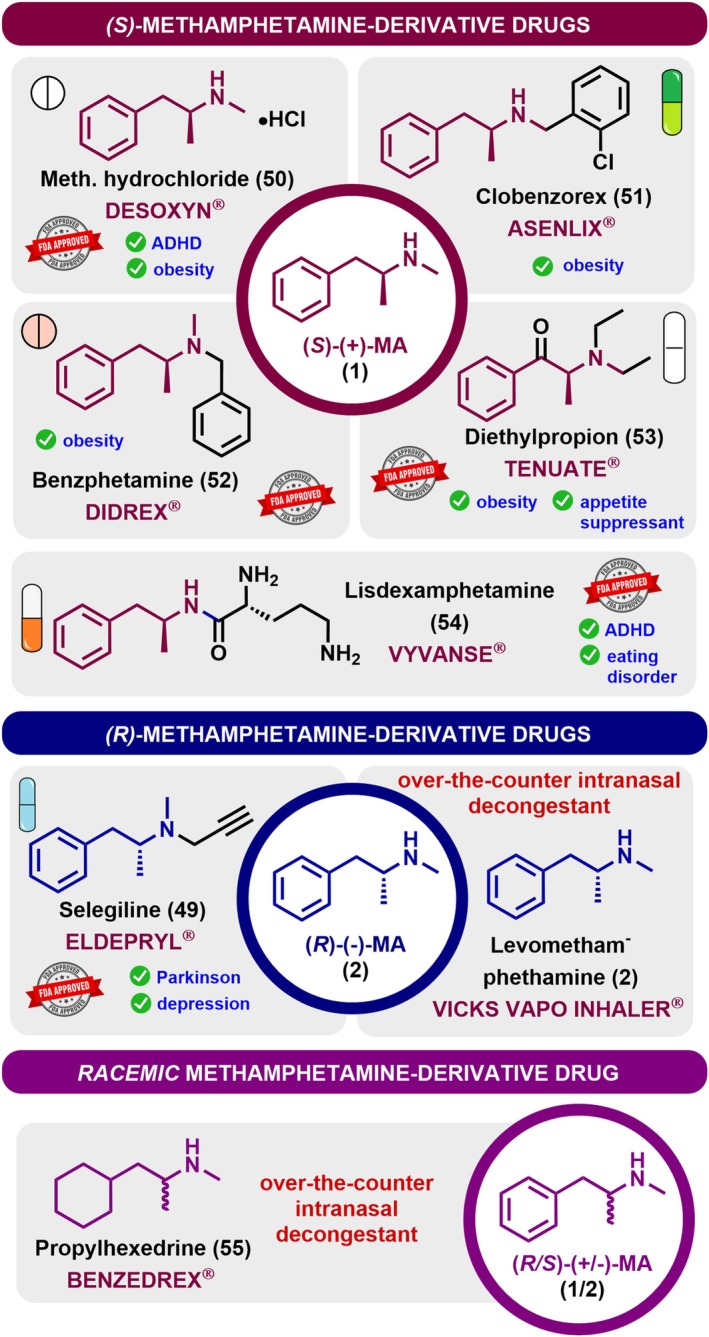
Therapeutic uses of both (*S*)‐(+)‐MA **(1)** and (*R*)‐(−)‐MA **(2)** derived medicinal drugs.

As previously discussed, MA exhibits multiple mechanisms of action, which differ significantly between its enantiomers. Indeed, there are medicinal drugs approved by the Food and Drug Administration (FDA) regulatory agency (Figure 4) derived from either (*S*)‐(+)‐ or (*R*)‐(−)‐MA (**1** or **2**, respectively) commercially available and prescribed for conditions such as attention deficit hyperactivity disorder (ADHD), obesity, and Parkinson's disease. The enantiomeric distinction is fundamental in shaping pharmacological outcomes: although both (*S*)‐(+)‐ and (*R*)‐(−)‐MA (**1** or **2**, respectively) share a common phenethylamine backbone, subtle stereochemical differences markedly affect CNS stimulation, cardiovascular effects, metabolism, and abuse potential [7].

## 9 Clinically Relevant Derivatives of (*S*)‐Methamphetamine

Due to its unfavorable risk profile, (*S*)‐(+)‐MA **(1)** itself is not marketed as a direct therapeutic agent, although its hydrochloride salt **50** (Desoxyn) is FDA‐approved for limited clinical use. This medicine, based on (*S*)‐methamphetamine hydrochloride **50**, is primely indicated for the treatment of ADHD and exogenous obesity [120]. However, its medical use is exceedingly rare today, having been largely supplanted by safer alternatives, such as methylphenidate [121].

Clobenzorex (**51**, Asenlix) was historically marketed in Mexico and parts of Latin America for obesity treatment [122]; however, it was never approved by the FDA. It undergoes metabolic conversion to AT and MA derivatives, which account for its pharmacological effects, but safety concerns (including false‐positive results in AT drug screening) led to its withdrawal from several markets worldwide [122].

A closely related compound, benzphetamine (**52**, Didrex) was approved by the FDA as an anorectic agent [123]. It functions as a prodrug that, similarly to Asenlix, is metabolized in vivo to active MA and AT derivatives [52]. Compared to Desoxyn, Didrex carries a lower abuse liability [124]. Nonetheless, its use is limited to short‐term weight management, and it is no longer widely marketed in the United States [125].

Following Desoxyn, several structural derivatives of (*S*)‐(+)‐MA **(1)** were developed to retain beneficial pharmacodynamic properties while reducing the risk of abuse. One notable example is diethylpropion (**53**, Tenuate), an FDA‐approved sympathomimetic agent used as a short‐term adjunct in the management of exogenous obesity [126].

Though not a direct MA derivative, lisdexamfetamine (**54**, Vyvanse) is included in this context as a prodrug of (*S*)‐(+)‐AT [127]. It was approved by the FDA for the treatment of ADHD in children aged 6 to 12 years, as well as adults and moderate‐to‐severe binge eating disorder [128]. As a polar, inactive prodrug, lisdexamfetamine requires enzymatic cleavage by red blood cells to release active (*S*)‐(+)‐AT gradually. This rate‐limited hydrolysis results in a smoother plasma concentration profile, characterized by a delayed onset and prolonged duration of therapeutic action. It significantly mitigates peak‐to‐trough fluctuations and reduces abuse potential via nonoral routes compared with immediate‐release stimulant medications.

## 10 Clinically Relevant Derivatives of (*R*)‐Methamphetamine

Unlike the (*S*)‐(+)‐MA enantiomer **(1)**, (*R*)‐(−)‐MA **(2)** exhibits markedly lower CNS activity; therefore, it has been therapeutically exploited to achieve stimulant‐like benefits with reduced abuse liability. A key clinical derivative is selegiline (**49**, Eldepryl), a selective and irreversible monoamine oxidase B (MAO‐B) inhibitor partially metabolized to (*R*)‐(−)‐MA **(2)** and (*R*)‐(−)‐AT [129, 130]. This medicine represented the first selective MAO‐B inhibitor and a major advance in Parkinson's disease therapy by prolonging dopaminergic activity [131]. Its transdermal formulation (Emsam), approved in 2006 for major depressive disorder [132], offers antidepressant efficacy with fewer dietary restrictions than oral MAO inhibitors [133].

Levomethamphetamine **(2)**, the active ingredient in the over‐the‐counter Vicks VapoInhaler, serves as a topical nasal decongestant through peripheral adrenergic vasoconstriction, providing symptom relief with minimal CNS stimulation [48].

Additionally, propylhexedrine **(55)** (Benzedrex), a racemic cycloalkylamine structurally related to MA, acts as an intranasal decongestant [134], with peripheral sympathomimetic effects similar to levomethamphetamine **(2)** [135]. Although case reports described misuse via oral or intravenous administration leading to AT‐like intoxication [136, 137], its continued over‐the‐counter availability reflects a balance between therapeutic utility and regulatory caution.

## 11 Conclusion

The comparative analysis of MA enantiomers underscores the decisive role of molecular chirality in governing pharmacological activity. A single stereocenter profoundly influences pharmacodynamics, pharmacokinetics, and overall physiological outcomes. The (*S*)‐(+)‐enantiomer accounts for MA's potent psychostimulant and addictive effects, whereas the (*R*)‐(−)‐form exhibits substantially weaker and distinct pharmacological behavior. Advances in enantioselective and stereocontrolled syntheses are therefore critical for isolating individual enantiomers and elucidating their biological specificities. Integrating chemical and biological insights refines our understanding of MA's structure–activity relationships and highlights the broader significance of chirality in drug design, toxicology, and therapeutic development.

## Data Availability

The data that support the findings of this study are available from the corresponding author upon reasonable request.
